# Functional Diversity of Homologous Oxidoreductases—Tuning of Substrate Specificity by a FAD-Stacking Residue for Iron Acquisition and Flavodoxin Reduction

**DOI:** 10.3390/antiox12061224

**Published:** 2023-06-06

**Authors:** Marta Hammerstad, Anne Kristine Rugtveit, Sondov Dahlen, Hilde Kristin Andersen, Hans-Petter Hersleth

**Affiliations:** Department of Biosciences, Section for Biochemistry and Molecular Biology, University of Oslo, P.O. Box 1066, Blindern, NO-0316 Oslo, Norway

**Keywords:** flavodoxin reductase, iron uptake, oxidoreductase, flavoenzyme, redox protein, protein interactions

## Abstract

Although bacterial thioredoxin reductase-like ferredoxin/flavodoxin NAD(P)^+^ oxidoreductases (FNRs) are similar in terms of primary sequences and structures, they participate in diverse biological processes by catalyzing a range of different redox reactions. Many of the reactions are critical for the growth, survival of, and infection by pathogens, and insight into the structural basis for substrate preference, specificity, and reaction kinetics is crucial for the detailed understanding of these redox pathways. *Bacillus cereus* (*Bc*) encodes three FNR paralogs, two of which have assigned distinct biological functions in bacillithiol disulfide reduction and flavodoxin (Fld) reduction. *Bc* FNR2, the endogenous reductase of the Fld-like protein NrdI, belongs to a distinct phylogenetic cluster of homologous oxidoreductases containing a conserved His residue stacking the FAD cofactor. In this study, we have assigned a function to FNR1, in which the His residue is replaced by a conserved Val, in the reduction of the heme-degrading monooxygenase IsdG, ultimately facilitating the release of iron in an important iron acquisition pathway. The *Bc* IsdG structure was solved, and IsdG-FNR1 interactions were proposed through protein–protein docking. Mutational studies and bioinformatics analyses confirmed the importance of the conserved FAD-stacking residues on the respective reaction rates, proposing a division of FNRs into four functionally unique sequence similarity clusters likely related to the nature of this residue.

## 1. Introduction

Homodimeric bacterial thioredoxin reductase (TrxR)-like ferredoxin/flavodoxin NAD(P)^+^ oxidoreductases (FNRs) are sequence-wise and structurally very similar to TrxRs, and even though many were originally annotated as TrxRs, they lack the TrxR catalytic redox-active CXXC-motif/Cys-pair responsible for the reduction of the thioredoxin (Trx) substrate [1]. Moreover, although FNRs commonly catalyze the electron transfer between NAD(P)H and the Fe–S cluster of ferredoxin (Fd) or the FMN cofactor of flavodoxin (Fld) [2,3], the co-existence of multiple FNR homologs in certain bacterial species has suggested and demonstrated multiple functions for different FNRs during the last decades and that these enzymes can act on numerous substrates and participate in different redox pathways [4]. This variation in substrate specificity has been suggested to be tuned by subtle structural differences among FNRs, despite sharing a conserved overall structural architecture [4]. Alternatively, in bacteria encoding only one (or no) copy of FNR, a fine-tuned promiscuousness towards multiple substrates highlights how ubiquitous these redox enzymes might be, evolved to participate in multiple cellular processes. The Firmicute *Bacillus cereus* (*Bc*) encodes three FNRs belonging to the TrxR-like subfamily of FNRs, two Flds, and the Fld-like protein NrdI. In our previous studies, we established the role of *Bc* FNR2 as the endogenous electron donor of NrdI, and hence, a redox partner involved in the activation of the dimanganese cluster of class Ib ribonucleotide reductase (RNR) in *Bc* [5,6,7,8]. Our further investigations of the various FNR-Fld/NrdI redox pairs showed that FNR2 reduces Fld1, Fld2, and NrdI with the highest reduction rates as compared to *Bc* FNR1 and FNR3 [9]. It has later been shown that *Bc* FNR3, the poorest Fld/NrdI reductase, as well as the homolog from *Staphylococcus aureus* (*Sa*), function as bacillithiol disulfide (BSSB) reductases (Bdrs), a crucial role in the maintenance of the reduced bacillithiol (BSH) pool in Firmicutes using this low-molecular-weight (LMW) thiol as a defense mechanism to buffer the intracellular redox environment and counteract oxidative stress encountered by human neutrophils during infections [10,11]. *Bc* FNR1 shares a high sequence identity with FNR2 (41%). However, our previous steady-state kinetics studies showed that the turnover number of FNR1, as compared to FNR2, was 380-fold, >200-fold, and >10-fold lower in the reduction of Fld1, Fld2, and NrdI, respectively [8,9]. Sequence and structural analysis of *Bc* FNR1 and FNR2 revealed that an aromatic residue, His, on the C-terminal helix stretching over the cofactor in the opposite monomer of the FNR dimer is conserved in FNR2s but is replaced by a conserved Val in several FNRs, including *Bc* FNR1, dividing these two FNRs into two phylogenetic clades of TrxR-like FNRs [9]. Although mutational studies of the FAD-stacking aromatic residue have suggested a shielding role of FAD from exposure to solvent during catalysis [12,13], none of these studies have mutated the aromatic residue to a hydrophobic, aliphatic residue such as Val. The interchange of the FAD-stacking residues might explain the large differences in the efficiency of Fld reduction by FNR1 and FNR2 and that the FNR1-type class, where Val is positioned on the FAD re-face, might prefer a different endogenous substrate than Fld.

The biological function of *Bc* FNR1 is unknown, although it has previously been shown to reduce Flds and NrdI at low efficiencies. An FNR1 homolog from *Sa*, named IruO (iron utilizing oxidoreductase), has been reported to contribute to iron release through the reduction of the heme-degrading monooxygenases IsdG and IsdI [14] and through the reduction of hydroxamate-type siderophores [15]; however, this function has only been reported for *Sa* IruO. The degradation of heme to staphylobilin and the subsequent release of ferrous iron is required by this pathogen during infection, and the electron supply from an oxidoreductase such as IruO plays a crucial role in the pathway [14,16]. This iron acquisition pathway may be a potential target for therapies in several pathogenic bacteria and underlines the importance of investigating possible oxidoreductases involved in iron acquisition further, in detail, and across species. The link between FNRs and their redox partners and the ferric uptake regulator (Fur) in terms of their versatility and roles in iron acquisition, as well as biological function in *Bc* and other Firmicutes, is an additional important aspect to examine when mapping or distinguishing the role of FNRs involved in iron acquisition.

In order to investigate the evolutionary divergence and functional variation among the structurally related FNRs in *Bc*, we investigated if FNR1 has a role in iron acquisition. Additionally, cross-mutants of the Val and His FAD-stacking residues in FNR1 and FNR2 were prepared to further investigate their potential effects on substrate specificity and differences in reduction rates of various substrates through tuning of the redox properties of the two FNR subclasses. Our findings show that *Bc* FNR1 can function as an IruO in the reduction of IsdG, resulting in the breakdown of heme and release of ferrous iron, suggesting a universal role in iron acquisition for the FNR1-type FNRs. Moreover, our mutational studies reveal a lowered reduction rate towards Flds and NrdI by FNR2, or towards IsdG by FNR1, when the His and Val FAD-stacking residues were interchanged, respectively, confirming the importance of these conserved residues stacking the FAD cofactor in the reduction of these specific substrates. Our study reveals a functional difference and promiscuity among these homologous enzymes from the same organism, largely influenced by the nature of the FAD-stacking residue and further underpinned by interesting structural aspects. The presented findings advance the detailed understanding of TrxR-like FNRs with respect to substrate specificity and extend the need for further characterization of functionally unique and specialized FNR classes.

## 2. Materials and Methods

### 2.1. Expression and Purification of Proteins

pET-22b(+) plasmids containing genes for FNR1 (BC0385), FNR1V329H (BC0385), FNR2H326V (BC4926), and IsdG (BC4542) (constructed using NdeI/HindIII sites), genes for Bdr (BC1495) and Fld2 (BC3541) (constructed using NdeI/BamHI sites), and the gene for NrdI (BC1353) (constructed using XbaI/HindIII sites) were ordered from GenScript Biotech, Rijswijk, Netherlands. Genes for FNR2 (BC4926) and Fld1 (BC1376) were cloned as described previously [8,17]. pET-22b(+) plasmids containing all the respective genes were transformed into competent *Escherichia coli* One Shot^TM^ BL21 (DE3) cells (Invitrogen, Thermo Fischer Scientific, Oslo, Norway), *E. coli* BL21 (DE3) cells (Novagen, Merck Life Science, Oslo, Norway), or *E. coli* BL21 Gold (DE3) cells (Novagen) for gene expression. Cells containing any of the nine plasmids were grown in a Terrific Broth medium containing 100 μg/mL ampicillin. Protein expression was induced by adding isopropyl β-D-thiogalactoside (IPTG) to a final concentration of 0.5–1.0 mM at OD_600 nm_ = 0.7–1.0, and the cultures were incubated for 12–18 h at 18–20 °C with vigorous shaking before cells were harvested and frozen at −20 °C. Cells were lysed and protein was purified as previously described for Fld1 and Fld2 [9], Bdr [10], NrdI [18], and FNR2 [8]. FNR1 and FNR1V329H [8,19] and FNR2H326V [8] were purified as previously described, with some deviations. Briefly, cells were thawed and dissolved in 100 mM Tris-HCl, pH 7.5, 1 mM DTT, and 5 μg/mL DNase, cOmplete Protease Inhibitor Cocktail (Roche, Oslo, Norway), in a 1:4 cell wet weight to buffer ratio and lysed by sonication. For FNR1 and FNR1V329H, contaminant proteins were precipitated with 0.3 and 0.23 g/mL ammonium sulfate ((NH_4_)_2_SO_4_), respectively. In contrast, the proteins of interest were precipitated by adding (NH_4_)_2_SO_4_ to the remaining supernatants to final concentrations of 0.5 and 0.4 g/mL (NH_4_)_2_SO_4_ for FNR1 and FNR1V329H. FNR2H326V was precipitated with (NH_4_)_2_SO_4_ to a final concentration of 0.2 g/mL (NH_4_)_2_SO_4_. Proteins were dissolved in 50 mM Tris-HCl, pH 7.5, and 1 mM DTT (FNR1 and FNR1V329H) or 50 mM Tris-HCl, pH 7.5, and 2 mM TCEP (FNR2H326V) and desalted using a HiTrap desalting column (Cytiva, Uppsala, Sweden) (FNR1V329H and FNR2H326V) or through dialysis (SnakeSkin™ dialysis tubing, 10K MWCO, ThermoFisher Scientific). Desalted proteins were applied to a HiTrap HP Q anion exchange column (Cytiva) and eluted with linear 0.1–0.45 M KCl, 0.18–0.4 M KCl, and 0–0.4 M KCl gradients for FNR1, FNR1V329H, and FNR2V326H, respectively. As a final polishing step, proteins were purified on a Superdex 200 Increase 10/300 GL column or Superose 12 10/300 GL column (Cytiva) in 50 mM Hepes, pH 7.5, and 100 mM NaCl (FNR1 and FNR1V329H) or in 50 mM Hepes, pH 7.5, 100 mM KCl, and 2 mM TCEP (FNR2V326H). For IsdG, contaminant proteins were precipitated with 0.15 g/mL (NH_4_)_2_SO_4_, whereas IsdG was precipitated with a final concentration of 0.35 g/mL (NH_4_)_2_SO_4_. Protein was dissolved in 50 mM Tris-HCl, pH 7.5, desalted through dialysis as described above, applied on a HiTrap HP Q anion exchange column (Cytiva), and collected using isocratic elution of the non-binding fraction with 50 mM Tris-HCl, pH 7.5. IsdG was purified on an XK16/70 column packed with Superdex 75 resin (Cytiva) and finally on a Superdex 75 Increase 10/300 GL column (Cytiva) in 50 mM Tris-HCl, pH 7.5, and 100 mM KCl. All chromatographic steps were performed using an Äkta purifier FPLC system (GE Healthcare, Oslo, Norway). Protein fractions were pooled, concentrated in Amicon Ultra-15 filter units (10 or 30 kDa MWCO, Merck-Millipore, Oslo, Norway), flash-frozen in liq N_2_, and stored at −80 °C. UV-visible (UV-vis) spectra were recorded, and the concentrations of proteins were estimated using extinction coefficients at their corresponding λ_max;_ ε_461/459_ = 10 mM^−1^ cm^−1^ for Fld1/Fld2 [20], ε_447_ = 10.8 mM^−1^ cm^−1^ for NrdI [8], ε_462/468_ = 9.9 mM^−1^ cm^−1^ for FNR1/FNR1V329H [8], ε_469/460_ = 11.1 mM^−1^ cm^−1^ for FNR2/FNR2H326V [8], ε_453_ = 11.5 mM^−1^ cm^−1^ for Bdr [8], ε_280_ = 17 mM^−1^ cm^−1^ for IsdG_apo_ (estimated with ProtParam, Expasy [21]), and ε_403_ = 148 mM^−1^ cm^−1^ for IsdG_heme_ [22], using an Agilent 8453 diode-array or an Agilent Cary 60 UV-visible spectrophotometer (Agilent Technologies, Santa Clara, CA, USA).

### 2.2. Reconstitution of IsdG with Hemin

Hemin (Sigma-Aldrich, Oslo, Norway) was dissolved in DMSO to a final concentration of 25 mM. For the crystallization of IsdG, hemin and IsdG were mixed in a 1:1 molar ratio and incubated for 1 h at 4 °C. Excess hemin and DMSO were removed using a Micro Bio-Spin 6 size-exclusion column (Bio-Rad, Oslo, Norway) equilibrated with 50 mM Tris-HCl, pH 7.5, and 100 mM KCl. For activity measurements, 25 mM hemin dissolved in DMSO was diluted 4× in 50 mM Hepes, pH 7.5, and 50 mM KCl (6.25 mM), added in a 1:1 molar ratio to IsdG, and allowed to bind for 1 h at 4 °C.

### 2.3. Protein Crystallization

All initial crystallization screening was performed with a Mosquito crystallization robot (SPT Labtech, Hertfordshire, UK). Conditions that identified initial hits were further optimized by systematic optimization using the sitting drop vapor diffusion method.

FNR2H326V crystals (12 mg/mL) were obtained with condition H11 from the Morpheus crystallization screen (Molecular Dimensions, Rotherham, UK) (0.02 M DL-glutamic acid monohydrate, 0.02 M DL-alanine, 0.02 M glycine, 0.02 M DL-lysine monohydrochloride, 0.02 M DL-serine, 20% *v*/*v* glycerol, 10% *w*/*v* PEG 4000, 0.1 M Tris/bicine, and pH 8.5). FNR1V329H crystals (39 mg/mL) were obtained with condition G8 from the Morpheus crystallization screen (Molecular Dimensions) (0.02 M sodium formate, 0.02 M ammonium acetate, 0.02 M sodium citrate tribasic dihydrate, 0.02 M potassium sodium tartrate tetrahydrate, 0.02 M sodium oxamate, 12.5% *v*/*v* MPD, 12.5% PEG 1000, 12.5% *w*/*v* PEG 3350, 0.1 M Hepes-Na/MOPS, and pH 7.5). IsdG_apo_ crystals (23 mg/mL) were obtained with condition D4 from the Morpheus crystallization screen (Molecular Dimensions) (0.02 M 1,6-hexanediol, 0.02 M 1-butanol, 0.02 M 1,2-propanediol, 0.02 M 2-propanol, 0.02 M 1,4-butanediol, 0.02 M 1,3-propanediol, 12.5% *v*/*v* MPD, 12.5% PEG 1000, 12.5% *w*/*v* PEG 3350, imidazole/MES monohydrate, and pH 6.5). Crystals of IsdG_heme_ were obtained with condition D8 from the Morpheus crystallization screen (Molecular Dimensions) (0.02 M DL-glutamic acid monohydrate, 0.02 M DL-alanine, 0.02 M glycine, 0.02 M DL-lysine monohydrochloride, 0.02 M DL-serine, 20% *v*/*v* glycerol, 10% *w*/*v* PEG 4000, Hepes-Na/MOPS, and pH 7.5). All crystals were grown at room temperature and flash-frozen in liquid N_2_ prior to data collection.

### 2.4. Crystal Data Collection and Processing

Diffraction data for FNR2H326V and FNR1V329H were collected at beamline ID30B at the European Synchrotron Radiation Facility (ESRF), Grenoble, France. Diffraction data for IsdG_heme_ and IsdG_apo_ were collected at beamline BioMAX at MAX IV, Lund, Sweden. All diffraction data were indexed and integrated through auto-processing with autoPROC [23] and XDS [24] and scaled and merged with Aimless in the CCP4 package [25].

#### 2.4.1. FNR2H326V

The structure was solved with molecular replacement (MR) with PHASER [26] using three search models—one monomer of *Bc* FNR2 (PDBid:6GAS), as well as two individual domains from the same structure, a FAD-binding domain and a NAD(P)H-binding domain. Initial refinements were performed using restrained refinement in REFMAC5 [27] followed by several cycles of refinement with phenix.refine [28] in the Phenix suite [29] and model building in Coot [30]. FNR2H326V was refined to 4.2 Å. The dimer is composed of an asymmetric architecture, with one FAD-bound monomer and one FAD-free monomer. The C-terminal subdomain of the FAD-free monomer stretches over the FAD-cofactor of the FAD-bound monomer, but not vice versa, and, hence, residues 318/319-331 could not be modeled for the latter.

#### 2.4.2. FNR1V329H

The structure was solved with molecular replacement using PHASER [26] and *Bc* FNR1 (PDBid:6GAR) as a search model. Refinement and model building was performed as for FNR2V326H. FNR1V329H was refined to 2.6 Å. The V329H mutation was clearly observed in the electron density. Residues 4–349 and FAD were included in both monomers.

#### 2.4.3. IsdG_heme_ and IsdG_apo_

The IsdG_apo_ structure was solved with molecular replacement using PHASER [26] and *Sa* IsdG (PDBid:1XBW) as a search model and IsdG_heme_ from the solved structure of IsdG_apo_. The structures contain two IsdG molecules in asymmetric units. Refinement and model building was performed as described above. For IsdG_heme_, the PDB-REDO server was also used [31]. IsdG_apo_ was refined to 1.9 Å and IsdG_heme_ to 2.0 Å. The IsdG_heme_ crystals showed a clear red-brownish color, and the electron density showed partial density for the heme group. Heme groups were therefore added to both monomers using the heme coordinates from PDBid:2ZDO as a starting point for refinement. The final heme group occupancies were set to 50%.

For all structures (FNR2H326V, FNR1V329H, IsdG_apo_, and IsdG_heme_), model validation was performed using MolProbity [32]. The absorbed X-ray dose was calculated using the program RADDOSE-3D [33]. All structure figures were prepared using PyMOL version 2.5 (Schrödinger, LLC, Mannheim, Germany).

### 2.5. Bioinformatics Analysis—Sequence Similarity Networks

Sequence similarity networks (SSNs) were generated with the Web-based Enzyme Function Initiative–Enzyme similarity tool EFI-EST (https://efi.igb.illinois.edu/efi-est/, accessed on 20 April 2023) [34], using *Bc* FNR1 as a search sequence in order to analyze FNR families with different residues stacking on the re-face of the FAD cofactor. The search was limited to a bacterial taxonomy group using the UniRef90 sequence database, retrieving 9020 homologous sequences grouped with an alignment score of 100 and nodes representing sequences that share >55% identity, dividing FNR1 and FNR2 into separate clusters. Figures illustrating SSN analyses were created in Cytoscape (version 3.9) with the organic layout [35]. Clusters containing more than 20 FNR sequences or 50 TrxR sequences were selected. FNRs containing His, Val, Tyr, or Phe in the FAD stacking position were identified and grouped into distinct clusters and compared to four clusters of TrxRs (CXXC-motif). For each cluster, a multiple sequence alignment was performed with Clustal Omega [36] through Jalview [37]. Consensus sequences of the residues stacking the FAD were generated with Weblogo [38].

### 2.6. Investigation of Potential Fur Control in Bc

Searching the RegPrecise Database [39] and manual inspection of the upstream sequences of selected genes for a Firmicute Fur-binding consensus sequence was performed to examine Fur regulation of selected FNR and IsdG genes in *Bc*, *Ba*, *Bs*, and *Sa*.

### 2.7. Protein–Protein Docking of FNR1-IsdG

Possible three-dimensional models of IsdG-FNR protein–protein complexes were generated using the ClusPro 2.0 server [40,41,42,43] and the LZerD protein docking server [44,45]. The IsdG-dimer (PDBid:8AVI) was used as a ligand, and the FNR1-dimer (PDBid:6GAR) and FNR2-dimer (PDBid:6GAS) were used as receptors. The structure of the FNR2-dimer was chosen due to its more open orientation of the NAD(P)H-binding domain relative to the FAD-binding domain, proposed to facilitate the binding of a larger ligand close to the FAD group. Furthermore, a truncated version of FNR1 with a deleted C-terminal subdomain (residues 321–347) was also included as a receptor in the docking studies, as the C-terminal tail shielding the FAD cofactor has been suggested to move during substrate binding. Selected top energetic hits from these runs placing the heme-Fe (IsdG) and FAD-N5 (FNR) groups within 25 Å apart were selected for inspection.

### 2.8. Activity Measurements

#### 2.8.1. Steady-State Kinetics of NrdI and Fld1 Reduction by FNR2H326V, and NrdI and Fld2 Reduction by FNR1V329H

The reduction of NrdI, Fld1, and Fld2 by NAD(P)H-dependent FNR2H326V and FNR1V329H was investigated through spectroscopic determination using an Agilent 8453 diode-array UV-visible (UV-vis) spectrophotometer. Due to the substrate’s high reactivity with dioxygen, experiments were carried out in a glove box (Plas Labs 855-AC, Lansing, MI, USA) under strict anaerobic conditions (91% N_2_, 9% H_2_, and Nippon gases, Oslo, Norway). All solutions were degassed on a Schlenk line before transfer to the glovebox. Buffers and stock solutions were sparged with argon for minimum two hours in vented vials, and protein samples were subjected to five to six cycles of evacuation before refilling with argon. All reactions were performed using 50 mM Hepes and 50 mM KCl buffer with pH 7.5 with constant stirring, and the temperature in the UV-vis cell was controlled with a temperature control accessory (Agilent, Santa Clara, CA, USA, 89090A) at 25 °C. Steady-state kinetic parameters for FNR2H326V and NrdI or Fld1 and steady-state kinetic parameters for FNR1V329H and NrdI or Fld2 were determined. Reduction was monitored by the disappearance of the NrdI_ox_ state with λ_max_ = 447 nm and appearance of the NrdI_sq_ state with λ_max_ = 575 nm, the disappearance of the Fld1_ox_ state with λ_max_ = 461 nm and appearance of the Fld1_sq_ state with λ_max_ = 594 nm, the disappearance of the Fld2_ox_ state with λ_max_ = 459 nm and appearance of the Fld2_sq_ state with λ_max_ = 592 nm, and monitoring the consumption of NADPH by a decrease at λ_max_ = 340 nm. All reactions were carried out with 200 μM NADPH, 0.5 μM enzyme, and various concentrations of NrdI, Fld1, and Fld2. The initial reduction rates in μM min^−1^ were determined as described previously [8]. The initial reduction rates were plotted against substrate concentration, and the data plots for each experiment were fitted with the Michaelis–Menten function using Origin (OriginLab Corporation, Northampton, MA, USA).

#### 2.8.2. Reduction of IsdG and Heme Degradation by FNR1, FNR2, Bdr, FNR1V329H, and FNR2H326V

The ability of the different reductases to supply electrons to IsdG, leading to the degradation of heme and release of iron, was investigated and compared. The reduction of IsdG was monitored by the disappearance of the IsdG λ_max_ = 403 nm state (IsdG_heme_ Soret peak), in order to follow the reduction of heme-bound Fe(III). Simultaneously, the decrease in the heme-bound Fe(II) state (λ_max_ = 427 nm) was monitored to determine the rate of heme degradation. All reactions were carried out with 200 μM NADPH, 1 μM enzyme (FNR1, FNR2, Bdr, FNR1V329H, or FNR2H326V), and 10 μM of reconstituted IsdG in 50 mM Hepes and 50 mM KCl buffer with pH 7.5. NADPH and buffer were mixed, IsdG was added (time = 0), and the enzyme was added after 950 s to start the enzymatic reaction and left to incubate with stirring for another 1050 s. Spectra were recorded every second.

#### 2.8.3. Steady-State Kinetics of IsdG Reduction and Heme Degradation by FNR1

Steady-state kinetic parameters for FNR1 and IsdG were measured. Reduction of IsdG and breakdown of heme was monitored by the disappearance of the Soret peak at 403 nm and the breakdown of heme at 427 nm. All reactions were carried out with 200 μM NADPH, 0.5 μM FNR1, and various concentrations of reconstituted IsdG (0.5–9 μM). FNR1 was added after 60 s for each measurement, and IsdG was added after 120 s to start the reaction. Spectra were taken every sec for 600 s and recorded using an Agilent 8453 diode-array UV-vis spectrophotometer. The initial reduction rate in μM min^−1^ was determined by plotting the breakdown of heme as a function of time and estimating the initial slope. The amount of reduced IsdG and breakdown of heme was calculated from the absorbance at 403 nm at the time points where the build-up of ferrous iron stops and heme degradation starts, similar to the procedure described by Loutet et al. [14]. The initial reduction rates were plotted against IsdG concentration, and the data plot was fitted with the Michaelis–Menten function using Origin (OriginLab Corporation).

## 3. Results and Discussion

The structural and functional basis for versatility in substrate specificity among TrxR-like FNRs is only starting to be unveiled. *Bc* encodes three homologous FNRs, which makes it a useful model system for studying and differentiating the characteristic features of this class of enzymes. Here, we will answer several questions with respect to distinguishing features, substrate specificity, and promiscuity of these enzymes in *Bc* and more generally among Firmicute bacteria. Which distinctive structural features do these FNRs have? Can FNR1 function as an iron uptake oxidoreductase, and what about the other FNRs encoded by *Bc*? How would a cross-mutation of the FAD-stacking residues Val and His influence the structure and the functional properties of FNR1 and FNR2?

### 3.1. Comparison of TrxR-Like FNRs in Bc and Related Species

**Figure 1:**
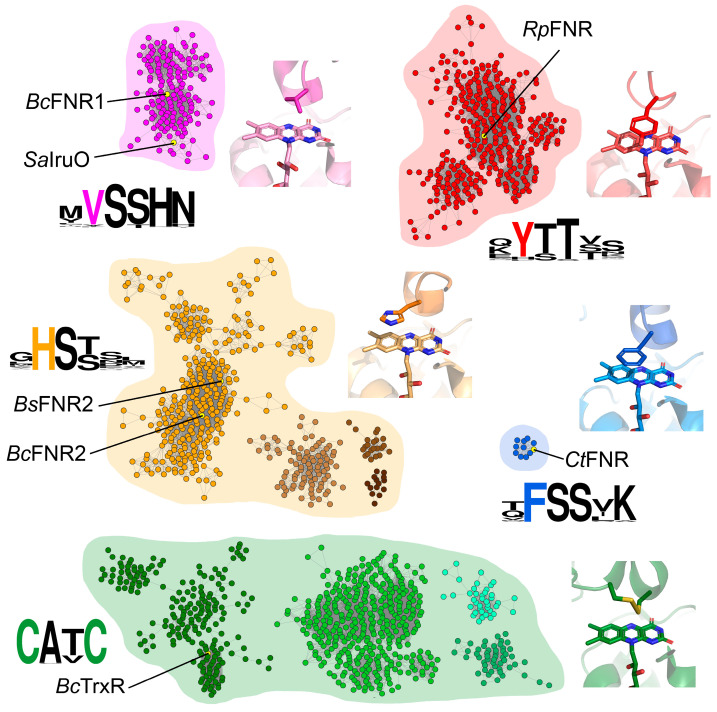
Comparison of TrxR-like FNRs through sequence similarity networks (SSNs). The SSN displays four clusters of FNRs, grouped according to their FAD-stacking residue (Val, His, Tyr, or Phe), and a cluster containing TrxRs (redox-active CXXC-motif). Examples of oxidoreductases representing each cluster with solved crystal structures are listed, and the crystal structures showing the vicinity of the FAD cofactor, including the conserved residue stacking on the *re*-face, are shown in corresponding colors: Val, *Bc*FNR1 (PDBid:6GAR); His, *Bc*FNR2 (PDBid:6GAS); Tyr, *Rhodopseudomonas palustris* FNR (*Rp*FNR) (PDBid:5YGQ); Phe, *Chlorobaculum tepidum* FNR (*Ct*FNR) (PDBid:3AB1); and CXXC, *Bc*TrxR (PDBid:7AAW). Monomers A are shown in a lighter shade than monomers B (TrxR, only one chain). Consensus sequences, including the conserved FAD-stacking residues, are shown for each cluster.

To further investigate and visualize the division of FNR1 and FNR2 into different functional groups and examine other functional trends across TrxR-like FNRs based on sequence similarity, SSNs were generated. Two groups of clusters resulted from the analysis using FNR1 as a search sequence, including a group containing TrxRs and four distinct groups containing TrxR-like FNRs lacking the CXXC-motif (Figure 1). The FNR groups of clusters could be categorized by the different residues stacking on the re-face of the FAD cofactor, namely His, Val, Tyr, or Phe (located on the opposite monomer of the dimer), as seen from the consensus sequences representing this region. For the different clusters identified, minimal variations are seen in residues located in the proximity of FAD, except for the characterized stacking residues listed above. In addition to these FAD-stacking residues, certain other differences among FNRs of different clusters can be observed, including sequence length, the length of the β-sheet hinge region connecting the two domains, and electrostatic surface potentials [9], which might influence the catalytic nature of these enzymes. Although structurally similar to FNR1, Bdrs are not clustered in any of the groups due to lower sequence identity (22%). Additionally, the latter enzymes are biological tetramers [10], and the FAD-stacking residue is not located on the C-terminal subdomain of the opposite monomer of the homodimer, as for the remaining FNR classes characterized in this study. These features likely distinguish Bdrs from the remaining FNRs in terms of dynamics of catalysis, tuning of the redox potential, and possibly regulation through cooperativity, as discussed below. The SSN supports the previous phylogenetic division of FNR1-like and FNR2-like FNRs [9] but further includes two additional SSN groups of FNRs containing Tyr and Phe as FAD-stacking residues. These findings highlight an important aspect that needs to be considered in the characterization of FNRs and an understanding of their growing substrate pervasiveness and modes of action.

### 3.2. Bc FNR1 Reduces IsdG

**Figure 2:**
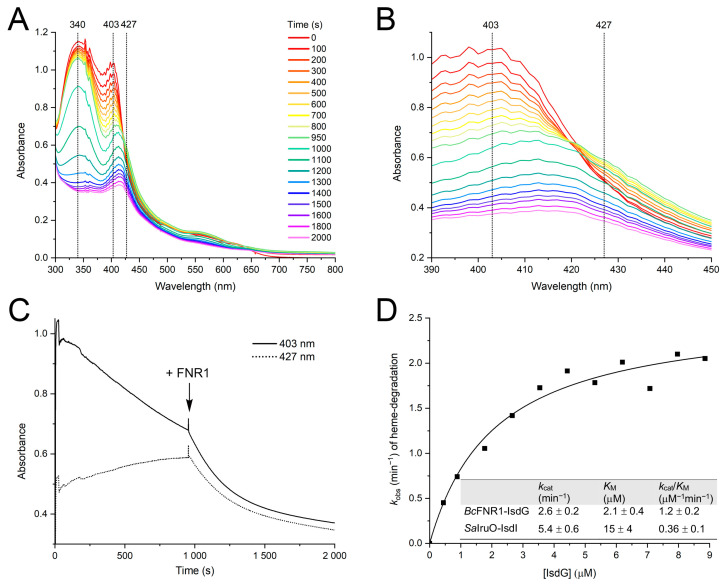
Reduction of IsdG and breakdown of heme by *Bc* FNR1. (**A**,**B**) UV-vis spectra showing the reduction of IsdG and subsequent degradation of heme following addition of FNR1 (950 s). Prior to the addition of enzyme, reduction of the heme-bound Fe(III) state (403 nm) and gradual accumulation of the heme-bound Fe(II) state (427 nm) is observed, as seen in (**B**,**C**). In contrast, breakdown of the heme-bound Fe(II) state is an FNR1-catalyzed reaction, only observed after the addition of enzyme. (**D**) Steady-state degradation of heme by FNR1, fitted with the Michaelis–Menten function, and steady-state kinetic parameters for the degradation of heme (IsdG) by FNR1 as well as steady-state parameters for the degradation of heme (IsdI) by *Sa* IruO [14]. For easier comparison, the parameters from [14] were converted to the same units as used for *Bc* in this study.

As *Bc* FNR1 and *Sa* IruO fall into the same SSN cluster, a general and specialized role in iron utilization could potentially be a key feature of oxidoreductases belonging to this group. Previously, IruO, a FAD-binding NAD(P)H-dependent oxidoreductase from *Sa,* has been described as an electron donor to the *Sa* heme-degrading proteins IsdI and IsdG [14], as well as to Fe(III)-hydroxamate-type siderophores [15]. Although the Isd-system and the role of IruO as an electron source have been characterized in *Sa*, no other studies have reported the function of a reductase used in iron acquisition in relation to the Isd system in other bacteria. TrxR-like FNR1 from *Bc* shares 45% of sequence identity with *Sa* IruO. Our previous studies investigated the role of *Bc* FNR1 as a reductase of Flds and the Fld-like protein NrdI. However, *Bc* FNR1 was shown to function as a significantly poorer reductase of Flds and NrdI as compared to the homologous FNR2 from *Bc* (>200-fold and >10-fold lower turnover number for the reduction of Fld2 [9] and NrdI [8], respectively). In this study, we demonstrate that the reduction of IsdG and IsdG-dependent heme degradation can be catalyzed by FNR1, as seen from our spectroscopic investigations after the addition of enzyme to a reaction mixture containing IsdG and NADPH (Figure 2). Prior to the addition of the enzyme and in the presence of NADPH, a reduction of the heme-bound Fe(III) state of IsdG is observed under aerobic as well as anaerobic conditions (latter data not shown). However, after the addition of FNR1 (after 950 s), the breakdown of heme is detected in the enzymatic reaction under aerobic conditions, confirming the importance of FNR1 for the degradation of heme in *Bc* IsdG and the resulting release of iron. From our analyses, it is clear that the decrease of the Soret peak at 403 nm is accelerated as compared to the non-enzymatic reduction of IsdG seen in the presence of NADPH (Figure 2A–C). Moreover, in the FNR1-catalyzed reaction, the decrease of the Soret peak detected at 427 nm demonstrates the breakdown of the heme-bound Fe(II) state, which is not observed for the non-enzymatic NADPH-driven reaction, where, instead, a build-up of heme-bound Fe(II) occurs.

**Figure 3:**
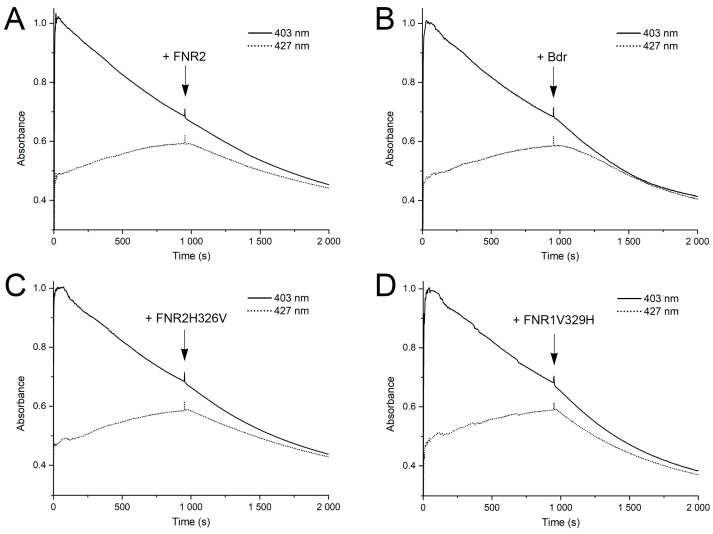
Reduction of IsdG and breakdown of heme by *Bc* (**A**) FNR2, (**B**) Bdr, (**C**) FNR2H326V, and (**D**) FNR1V329H, as monitored by UV-vis spectroscopy. For all reductases (**A**–**D**), the decrease of the Soret peak at 403 nm is slightly accelerated upon the addition of enzyme (950 s) as compared to the non-enzymatic reduction of IsdG, accompanied by a breakdown of the heme-bound Fe(II) state monitored at 427 nm. However, the rates of IsdG reduction and heme degradation are evidently slower than the FNR1-catalyzed reaction shown in Figure 2.

To determine steady-state kinetic parameters for FNR1 with IsdG, initial reduction rates were measured and plotted against the concentration of IsdG, and data were fitted with the Michaelis–Menten function (Figure 2D). The reaction between FNR1 and IsdG proceeds with a *k*_cat_ of 2.6 ± 0.2 min^−1^, the *K*_M_ of FNR1 for IsdG was determined to be 2.1 ± 0.4 μM, and the catalytic efficiency (*k*_cat_/*K*_M_) was 1.2 ± 0.2 μM^−1^min^−1^. Kinetic parameters for the reaction between *Sa* IruO and IsdG (37% sequence identity to *Bc* IsdG) have not been determined, as the increase in enzyme velocity was in the linear range for the substrate concentrations (1–25 μM) investigated by Loutet et al. [14]. However, in the same study, the kinetic parameters were determined for the IsdG paralog IsdI (35% and 64% sequence identity to *Bc* and *Sa* IsdG, respectively). Compared to the steady-state kinetic parameters reported for *Sa* IruO with IsdI, the turnover number of *Bc* FNR1 with IsdG is in the same order of magnitude (~2-fold higher for *Sa* IruO-IsdI), whereas the *K*_M_ of FNR1 for IsdG from *Bc* is ~7-fold lower than determined for *Sa* IruO-IsdI [14], leading to a ~3-fold higher catalytic efficiency for the reaction in *Bc* as compared to *Sa* (Figure 2D). For comparison, the breakdown of heme was investigated using *Bc* FNR2 and Bdr as potential reductases of IsdG, where both reductases showed a decreased rate of IsdG reduction and heme breakdown as compared to the FNR1-catalyzed reaction (2.5 to 7-fold slower reduction rates as compared to FNR1) (Figure 3A,B), indicating that these enzymes are poorer IruOs than the preferred FNR1.

**Figure 4:**
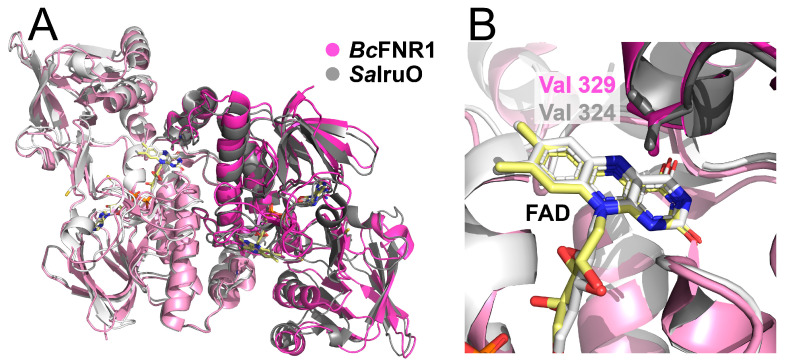
Alignment and comparison of *Bc* FNR1 (PDBid:6GAR) and *Sa* IruO (PDBid:5TWB) crystal structures. (**A**) The overall folds show a similar orientation of the NAD(P)H-binding domain relative to the FAD-binding domain, and (**B**) both reductases contain the conserved Val residue stacking on the *re*-face of the FAD group, located on the C-terminal subdomain of the opposite monomer of the dimer. Monomers A are shown in lighter shades than monomers B and FAD cofactors, and *Sa* IruO non-conserved Cys residues (Cys248 + Cys265) are shown as sticks and colored by atom type (*Bc* FNR1 FAD carbons colored yellow).

Based on our findings, we propose that FNR1-type FNRs, where Val is positioned on the FAD *re*-face, may function as electron donors to the heme-degrading monooxygenases IsdG and IsdI throughout Firmicutes encoding one or both of the latter monooxygenase paralogs. The structural alignment of *Bc* FNR1 (PDBid:6GAR) and *Sa* IruO (reduced form, PDBid:5TWB) shows an overall conserved fold, with the Val residues stacked opposite to the FAD cofactors (Figure 4). Moreover, both structures show a similar orientation of the NAD(P)H-binding domain relative to the FAD-binding domain. Both structures also contain a longer C-terminal helix than the FNR2-type FNRs, the latter containing a conserved His positioned on the FAD *re*-face (Figure 1). Rotation of the NAD(P)H-binding domain relative to the FAD-binding domain has been suggested to be important for substrate binding and catalysis in FNRs [4,9,12].

For *Sa* IruO, maintaining two cysteine residues in their reduced state was suggested to be important to avoid a more “closed” and covalently fixed domain conformation and hence a narrowing of the Fe(III)-siderophore substrate binding site [15]. However, as these cysteines are not conserved throughout homologs of IruO, such as *Bc* FNR1, the relevance of these residues in the regulation of IruO activity is likely restricted to limited IruOs and not of general importance for IruO activity.

### 3.3. Structure of IsdG and the Putative IsdG-FNR Complex

**Figure 5:**
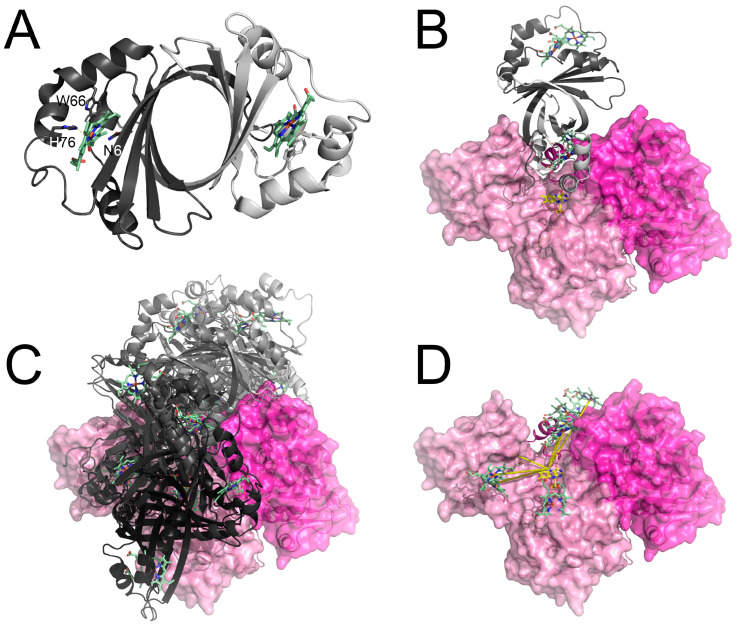
The crystal structure of *Bc* IsdG and docking models of *Bc* IsdG-FNR1 complex. (**A**) The structure of the *Bc* IsdG dimer, with the heme group and the conserved active site residues (His76, Trp66, and Asn6) required for catalytic activity shown as sticks and colored by atom type. (**B**) A selected docking model of the IsdG-FNR protein–protein complex, showing that IsdG can bind in the cleft between the FAD-binding domain and the NAD(P)H-binding domain, positioning the heme of IsdG adjacent to the FAD cofactor of FNR1. (**C**,**D**) Selected top hits of putative IsdG-FNR1 models placing the heme and FAD groups within a 25 Å distance, with IsdG shown as cartoon representation (**C**), or showing only heme groups from the corresponding IsdG structures (only monomers with heme closest to FAD) as sticks (**D**). Monomers A and B are colored in different shades.

The structure of *Bc* IsdG (Figure 5A and Table 1) determined in this study is highly similar to the structure of *Sa* IsdG and IsdI with an RMSD of 1.0 Å between *Bc* IsdG_apo_ and *Sa* IsdG (PDBid:1XBW). The IsdG monomers consist of Fd-like α/β-sandwich folds forming a β-barrel at the dimeric interface containing the conserved Asn-Trp-His triad required for the catalytic activity in the heme binding pockets [46]. *Bc* IsdG was crystallized in its apo-form and reconstituted with hemin, the latter yielding red-brownish crystals. The electron density for the heme groups of IsdG_heme_ was, however, relatively poor, and only 50% occupancy could be modeled, with apparent density for the iron ions but limited for the rest of the porphyrin. In addition to low occupancy, the heme groups might be bound with different orientations and degrees of ruffling [47,48].

As we have demonstrated that FNR1 can reduce IsdG, the putative interaction was investigated through protein–protein docking. The selected top hits with the shortest heme-Fe (IsdG) to FAD-N5 (FNR) distances cover a large potential binding area of FNR1 (Figure 5B–D). In contrast to our previous docking results for Fld to FNR1 and FNR2, it seems likely that IsdG can bind in the less exposed binding cleft found in the FNR1 structure, which is narrower than the more “open” conformation observed in the FNR2 structure, with the latter suggested to facilitate binding of Fld [9]. The docking of IsdG to multiple FNR1 sites, spreading across the binding site in a fan-like distribution, is in accordance with the interaction between cytochromes and methylamine dehydrogenase, where only an approximate orientation was needed for electron transfer [49].

### 3.4. Difference in Regulation of FNR1 and FNR2-Fur Control of Reductases Involved in Iron Acquisition in Firmicutes

Our enzymatic assays demonstrate that the IsdG heme degradation reaction proceeds slower using FNR2 or Bdr as compared to FNR1. Can the basis behind these differences be related to gene regulation? The expression of genes required for iron uptake is known to be regulated by the ferric uptake regulator Fur. Fur proteins function as iron-responsive repressors. Fe(II) limitations lead to the derepression of genes required for Fe(II) uptake, and Fe(II) excess induces the expression of genes involved in iron efflux [50,51]. The Fur protein is the most widespread bacterial iron sensor, critical for maintaining iron homeostasis, acting through the recognition of and binding to DNA sequences within the target promoters called Fur boxes [52,53,54,55]. Breakdown of heme and release of ferrous iron is part of one such iron acquisition strategy involving IsdG [46,56] and IruO in *Sa* [14], both being under the control of the Fur repressor, with Fur boxes identified upstream of the respective genes [14,57]. Similarly, we observe a 19-base pair (AATGATAATGATTATCACT) sequence upstream of the *Bc* FNR1 gene consistent with 18 of the bases with respect to the consensus Fur box of *Bacillus subtilis* [52,58].

**Figure 6:**
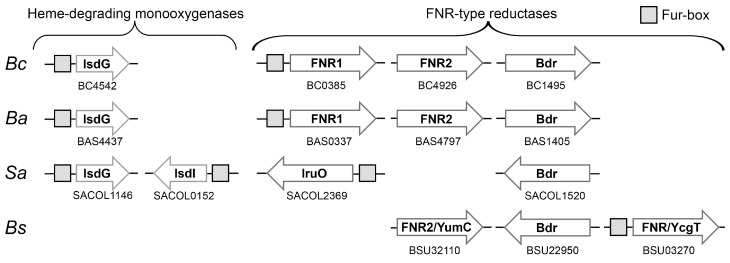
Schematic representation of loci of oxidoreductases and heme-degrading monooxygenase genes in selected Firmicutes, regulated by Fur. Among the genes encoding FNRs in *Bc*, *Ba,* and *Sa*, only FNR1-like FNRs (FAD-stacking Val residue) are proposed to be Fur-regulated, containing an upstream Fur box (gray square), and are likely involved in iron acquisition in these bacteria, unlike FNR2-type FNRs and Bdrs. *Bs* does not encode an FNR1-like FNR but instead encodes another Fur-regulated FNR, YcgT, sequence-wise similar to *Bc* FNR1 but with a Tyr residue stacking on the FAD *re*-face. Locus tags are listed under the corresponding protein names.

Hence, the likely Fur regulation of both IruO in *Sa* and FNR1 in *Bc* supports that both these reductases take part in iron acquisition pathways (Figure 6).

*Ba* encodes an FNR1-homolog as well, which also contains an upstream Fur box. Moreover, the three latter bacteria encode IsdG (and IsdI for *Sa*), suggesting a conserved interplay between IruO (FNR1) and IsdG. *Bc*, *Ba*, and *Sa* all encode the homologous Bdr shown to reduce BSSB, but with a low reduction rate of IsdG (Figure 3B), lacking the upstream Fur box, and not surprisingly, likely not involved in iron acquisition. *Bc* and *Ba* both encode the FNR2-type FNR with a His residue stacking the FAD cofactor, a poorer IsdG reductase (Figure 3A) shown to be involved in the activation of class Ib RNR [8] and also lacking a Fur box. These findings underpin the role of FNR1-type FNRs as IsdG reductases in bacteria encoding this group of FNRs. This is further strengthened by the fact that *Bs* does not encode an FNR1-type FNR (with Val stacking on the FAD re-face), nor IsdG, and instead uses other Fur-regulated systems for iron acquisition [52]. *Bs* does, however, encode a different Fur-regulated FNR, YcgT, with a Tyr residue stacking on the FAD re-face. Nonetheless, YcgT is sequence-wise more similar to FNR1 than the other members of the SSN-cluster containing Tyr on the FAD *re*-face. Although several mechanisms for iron uptake are used by *Sa*, *Bc*, and other Firmicutes, these findings and the co-existence of an IruO and IsdG in these bacteria suggest that the FNR1-type FNRs have evolved to function in the reduction of IsdG as part of an iron uptake strategy.

### 3.5. The Effect of the Conserved Val and His in FNR1 and FNR2 on Reduction Rates

From our previous studies and the results presented in this work, we have proposed that *Bc* FNR1 and FNR2 belong to two different functional groups of TrxR-like FNRs and can structurally be differentiated by the stacking residue on the *re*-face of the FAD group being Val or His, respectively [9]. Our previous phylogenetic analysis also showed that FNR2-type FNRs are found in Firmicutes, Proteobacteria, Actinobacteria, and green sulfur bacteria. In contrast, the FNR1-group consists solely of members of the Firmicutes phylum, indicating that this type of FNRs has evolved explicitly in Firmicute bacteria for a reason.

**Figure 7:**
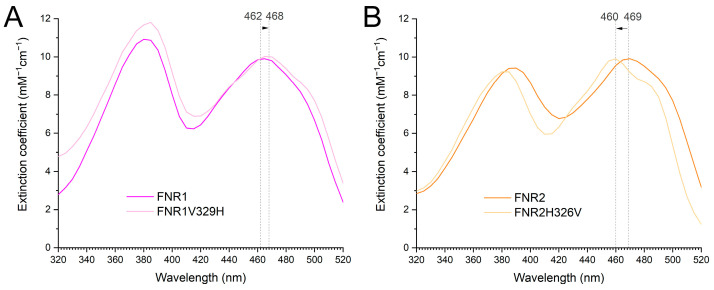
UV-vis spectra of the oxidized state of *Bc* FNR1 and FNR2 and the corresponding FNR1V329H and FNR2H326V mutants showing an upshift of the spectrum at λ_max_ caused by the V329H mutation in FNR1 (**A**) and a downshift of the spectrum at λ_max_ caused by the H326V mutation in FNR2 (**B**).

In this work, we have investigated the effect of the FAD-stacking residues on the electron transfer rates using different substrates assigned to the two FNRs—IsdG for FNR1 and Flds/NrdI for FNR2 [8,9]. To investigate if the interchange of Val to His in FNR1 and His to Val in FNR2 could explain the differences in the efficiencies of Fld or IsdG reduction, we generated cross-mutants of the two proteins, FNR1V329H and FNR2H326V. The effect of the mutations can be seen spectroscopically (Figure 7). The environment surrounding the FAD cofactor is clearly influenced in both mutants. For the FNR2H326V mutant, the λ_max_ peak is shifted to a lower wavelength, while for FNR1V329H, it is shifted to a higher wavelength.

The effects of the mutations were first investigated on the reduction of Flds and NrdI. In the case of FNR2H326V, the replacement of His with Val resulted in a nearly 3-fold decrease in the turnover number in the reduction of NrdI and a 15-fold decrease in the reduction rate of Fld1, as compared to our previous studies on the wild-type protein [8] (Table 2). However, the *k*_cat_ values for the reduction of NrdI and Fld1 determined in this study are higher for FNR2H326V than for FNR1, the latter containing a naturally occurring FAD-stacking Val residue, indicating that other structural aspects of FNR2, in addition to the role of the His residue, are important for the high turnover reported for FNR2 as a reductase of Flds and NrdI [8,9]. Nevertheless, these findings greatly support the importance of the aromatic FAD-stacking residue found in FNRs belonging to the FNR2-type subclass in the reduction of Flds. In the case of FNR1V329H, replacing Val with His did not lead to an increase in the turnover number in the reduction of NrdI; on the contrary, we observed a *k*_cat_ value 4-fold lower than for FNR1 and 55-fold lower than for FNR2 [8]. For Fld2, the turnover number using FNR1V329H as a reductase was shown to be 2-fold lower than with FNR1 and 480-fold lower than with FNR2 [9]. Although FNR1 has been previously shown to be a poorer reductase of Flds and NrdI than FNR2, these findings suggest that replacing the FAD-stacking Val residue for His in FNR1-type FNRs alone is not enough to make FNR1 a more efficient Fld reductase, somewhat distorting the fine-tuned chemical environment in the vicinity of the FAD cofactor. Our previous redox potential measurements substantiate the reduction kinetics and high electron transfer rates observed for FNR2, with an *E*_ox/sq_ (reduction potential for the FAD oxidized/semiquinone state) of −332 mV, as compared to an *E*_ox/sq_ of −228 mV for FNR1 [9].

Next, the effect of these cross-mutations in FNR1 and FNR2 was investigated on IsdG as a substrate by monitoring the breakdown of heme and comparing the results to the reactions catalyzed by wild-type FNRs and Bdr (Figure 2C and Figure 3). Comparing FNR1V329H to FNR1 as an IruO reveals that the FAD-stacking Val residue is a key structural feature important for the reduction of IsdG and breakdown of heme, as an approximately 2-fold slower rate of heme breakdown is observed using FNR1V329H as a reductase as compared to the wild-type enzyme. An even slower reduction rate of IsdG and breakdown of heme is observed using Bdr, FNR2, or FNR2H326V, with no significant differences observed for the wild-type FNR2 as compared to the mutant FNR2H326V. Together, these findings demonstrate that *Bc* FNR1/IruO serves as the most efficient reductase of IsdG and facilitates the breakdown of heme with the highest rate as compared to FNR2 and Bdr, whose biological roles have been established as reductases of Fld/NrdI [8,9] and BSSB [10,11], respectively. The presence of a Val residue stacking the FAD in FNR1 is essential for the higher reduction rate of IsdG, as shown in this study. In contrast, the introduction of such an aliphatic FAD-stacking residue in the FNR2H326V mutant did not appear to improve the ability to function as an IsdG reductase for FNR2 to a significant extent.

These findings demonstrate that the differences in electron transfer rates and substrate specificities of FNR1 and FNR2 (and likely other FNRs belonging to the same phylogenetic subclasses of TrxR-like FNRs) towards IsdG and Flds/NrdI as substrates, respectively, are partly tuned by the nature of the FAD-stacking residues, Val and His, conserved among members of these groups of FNRs. Our SSN studies show that conserved Tyr and Phe residues can substitute His and Val as residues interacting with the isoalloxazine ring of FAD in TrxR-like FNRs. Examples of such FNRs are FNR from the photosynthetic purple non-sulfur bacterium *Rhodopseudomonas palustris* (Tyr, PDBid:5YGQ) and FNR from photoautotrophic green sulfur bacteria, including *Chlorobaculum tepidum* (Phe, PDBid:3AB1) (Figure 1), which cluster in distinct clades. It has been shown that the FAD-stacking Tyr in *R. palustris* FNR enhances the release and re-association of NADP^+^/NADPH [59]. It is of interest, however, to further investigate whether these conserved FAD-stacking residues could be responsible for altering the substrate preference among FNRs and ultimately affecting and specializing their roles as reductases of distinct substrates, making the residues a vital link between structure and function, despite the weak and barely distinguishable binding affinities determined for the *Bc* FNR1/FNR2/Bdr-NrdI redox pairs, typically observed for electron transfer complexes [60]. However, other structural aspects and the sum of the catalytic environment, the size, flexibility, and properties of the substrate binding pocket, as well as the length and nature of the C-terminal helix [13,61,62], are likely involved in the control of and tuning of substrate specificity among these FNR classes.

### 3.6. The Crystal Structure of the FNR2H326V Mutant Can Explain the Lowered Reduction Rate towards Flds

To investigate whether the altered reduction rates observed for the FNR2H326V and FNR1V329H mutants could be explained by their crystal structures, both structures were solved (Table 1). The FNR1V329H structure is, with the exception of the mutation, highly similar to the wild-type FNR1 structure with an RMSD value of 0.2 Å. The mutation has not led to a change in the orientation of the NAD(P)H-binding domain relative to the FAD-binding domain, nor a movement of the other residues in the vicinity of the FAD cofactor. The lowered activity observed for FNR1V329H must therefore, from a structural perspective, be directly caused by the chemical properties of the single Val to His mutation, strengthening the importance of this residue for the biological function of FNR1.

**Figure 8:**
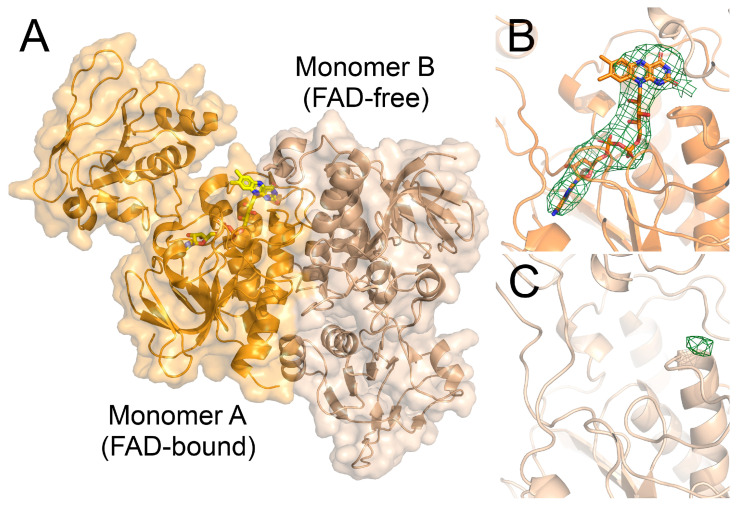
The *Bc* FNR2H326V crystal structure reveals structural alterations caused by replacing the FAD-stacking His residue with Val. (**A**) The structure consists of an asymmetrically arranged dimer, where the two monomers show different conformations of the NAD(P)H-binding domains relative to the FAD-binding domains. The FAD-bound monomer (orange) shows the same conformation as seen for the wild-type *Bc* FNR2 structure (PDBid:6GAS), whereas the second monomer (wheat), showing a different conformation, completely lacks the presence of the FAD cofactor. (**B**) The FAD cofactor bound in monomer A, shown as sticks and colored by atom type, surrounded by the omit map contoured at 3σ. (**C**) FAD binding site of monomer B, missing the FAD cofactor. The *F*_o_-*F*_c_ map is shown as green and red mesh, contoured at 3σ and −3σ, respectively.

In contrast, the FNR2H326V crystal structure revealed interesting and unusual features, including an asymmetric rotation of the NAD(P)H-binding domain relative to the FAD-binding domain in the two monomers of the biological dimer (Figure 8).

One of the monomers shows the same domain conformation as seen for the native *Bc* FNR2 crystal structure (PDBid:6GAS) and *Bs* FNR2 (YumC) (PDBid:3LZX), a favored conformation supporting binding of the Fld substrate, as proposed through docking studies [9]. Interestingly, the second monomer has adopted a different conformation of the NAD(P)H-binding domain, resulting in an asymmetric dimer. This conformation is more closed, resembling the low-molecular-weight (low *M*_r_) TrxR structure, where a different conformational change is required for catalysis than for FNRs [63]. Furthermore, the second monomer with the alternative conformation completely lacks electron density corresponding to the FAD cofactor and is, in fact, an apoprotein. In the conformation seen for the FAD-free monomer, the position conventionally occupied by FAD is not stacked by the C-terminal subdomain of the opposite monomer. Such an asymmetric structure of FNR, composed of two monomers with different conformations, has only been described once previously [12] and is not a common feature in homodimeric FNRs. Moreover, the total loss of the FAD cofactor in one monomer, and a 100% occupancy in the other has never been described previously.

These structural features caused by the replacement of the FAD-stacking His residue with Val in the FNR2H326V mutant may be the reason for the reduced reduction rate observed for FNR2H326V. However, the protein concentration was measured based on the FAD concentration using ε_469_ = 11.1 mM^−1^ cm^−1^, ensuring the correct total amount of FAD-bound polypeptides used for activity measurements. Therefore, the structural asymmetry and loss of FAD, caused by the mutation, could influence the reduction rate by rendering the FNRH326V dimer unstable or through an inhibiting effect caused by the FAD-free monomer. It is unknown, however, whether the mutation leads to the loss of FAD, ultimately affecting the conformation, or whether a conformational alternation triggers the loss of FAD. Additionally, the asymmetry in FNR2H326V indicates that the enzyme may exhibit a negative cooperativity with respect to the binding of FAD, possibly affecting the reaction rate of Fld reduction. A similar feature has also been observed for the binding of NADPH in *Bc* TrxR, indicating that dimeric TrxRs and TrxR-like FNRs can to some extent exhibit negative cooperativity features [64].

## 4. Conclusions

Our bioinformatics analyses through SSNs, as well as functional studies, demonstrate that TrxR-like FNRs can be divided into distinct functional groups based on their sequences and the nature of the conserved residue stacking the FAD cofactor. By investigating and comparing the redox properties of the different FNRs encoded by *Bc* with respect to substrate specificity, we have shown that *Bc* FNR1 can function in iron utilization by reducing *Bc* IsdG and facilitating the degradation of heme. We propose a universal role in this iron uptake strategy for FNR1-type FNRs from Firmicutes homologous to *Bc* FNR1, which contain a conserved Val residue stacking on the FAD *re*-face and are controlled by Fur. Mutational studies further underlined the importance of the Val residue in FNR1 for efficient heme degradation, as seen from the lower rate observed using the FNR1V329H mutant or other *Bc* TrxR-like FNRs. Vice versa, replacing the conserved FAD-stacking His residue found in FNRs belonging to the FNR2-type subclass with Val resulted in a decrease in the reduction rate of the endogenous substrate of *Bc* FNR2, further emphasizing the influence of these conserved residues on substrate specificity and catalytic efficiency. We suggest that FNR homologs belonging to the distinct SSN groups are assigned to specific biological processes in *Bc*, likely typifying other evolutionary-related FNRs throughout bacterial species as well. Our findings present an important contribution to the understanding of substrate specificity and mode of action in FNRs and draw further attention to the link between function and structure among these unique FNR classes.

## Figures and Tables

**Table 1:** Crystal Data Collection and Refinement Statistics.

	*Bc*IsdG_apo_	*Bc*IsdG_heme_	*Bc*FNR1V329H	*Bc*FNR2H326V
Data collection				
X-ray source	MAXIV BioMAX	MAXIV BioMAX	ID30B	ID30B
Detector	Eiger 16M	Eiger 16M	Pilatus 6M	Pilatus 6M
Wavelength (Å)	0.9700	0.9700	0.9763	0.9763
Space group	P2_1_2_1_2_1_	P2_1_2_1_2_1_	P2_1_2_1_2	P2_1_2_1_2_1_
*a*, *b*, *c* (Å)	46.4, 48.9, 101.7	46.7, 48.9, 100.9	164.6, 56.9, 94.4	71.9, 105.1, 216.9
α, β, γ (°)	90, 90, 90	90, 90, 90	90, 90, 90	90, 90, 90
Rotation	Standard	Standard	Standard	Standard
Rotation range per image (°)	0.20	0.20	0.05	0.10
Total rotation range (°)	180	180	100	110
Exposure time per image (s)	0.011	0.011	0.020	0.020
Flux (ph/s)/Transmission (%)	1.9 × 10^12^/100	2.4 × 10^12^/100	3.7 × 10^11^/1.7	1.4 × 10^12^/6.1
Beam size (µm^2^)	50 × 50	50 × 50	30 × 30	30 × 30
Crystal size (µm^3^)	200 × 50 × 30	150 × 70 × 30	50 × 30 × 20	30 × 25 × 25
Absorbed X-ray dose (MGy)				
- Av. diffraction weighted dose;	1.0	1.4	1.3	6.4
- Average dose (exposed regions);	1.2	2.0	2.5	12.9
- Max. dose.	2.5	3.5	3.1	13.2
Mosaicity (°)	0.22	0.27	0.06	0.22
Resolution range (Å)	42.22–1.90 (1.94–1.90)	44.04–2.00 (2.05–2.00)	53.81–2.60 (2.72–2.60)	68.21–4.20 (4.70–4.20)
Total no. of reflections	119,246	105,677	98,595	47,888
No. of unique reflections	18,723	16,227	26,835	11,894
*R*_meas_	0.070 (1.003)	0.071 (1.313)	0.100 (0.575)	0.202 (1.092)
*R*_merge_	0.064 (0.901)	0.065 (1.213)	0.087 (0.498)	0.177 (0.962)
Completeness (%)	99.1 (95.2)	99.7 (100.0)	96.1 (97.6)	95.8 (96.8)
Multiplicity	6.4 (5.2)	6.5 (6.7)	3.7 (3.6)	4.0 (4.2)
*<I*/σ(*I*)>	12.7 (1.8)	12.2 (1.6)	8.7 (2.2)	5.6 (1.7)
CC_1/2_	0.997 (0.556)	0.997 (0.579)	0.996 (0.848)	0.994 (0.552)
Refinement statistics				
*R*_work_/*R*_free_ (%)	19.7/23.6	20.1/23.9	18.8/23.8	22.7/31.1
Mean protein/cofactor/waters isotropic *B* factor (Å^2^)	46.4/-/46.5	59.9/87.9/56.6	81.8/53.8/53.2	186.2/168.8/-
Wilson B-factor (Å^2^)	37.8	47.7	55.8	157.8
Protein assembly in asymmetric unit (AU)	Homodimer	Homodimer	Homodimer	Asymmetric homodimer
Protein residues in gene	107	107	349	331
Total modelled residues in AU				
- Protein residues by chain;	A: 1–106, B: 1–106	A: 1–106, B: 1–106	A: 4–349, B: 4–349	A: 6–319, B:5–331, C: 6–318, D: 6–331
- Cofactors;	-	A: 1 heme, B: 1 heme	A: 1 FAD, B: 1 FAD	A: 1 FAD, C: 1 FAD
- Added waters.	82	42	59	0
Matthews coefficient (Å^3^/Da)	2.4	2.3	2.8	2.8
Solvent content (%)	48.5	45.9	55.9	56.1
Ramachandran favored/allowed/outliers (%)	97.1/2.9/0.0	97.1/2.4/0.5	96.7/3.2/0.2	90.6/8.8/0.6
RMSD bond lengths (Å)	0.007	0.008	0.008	0.004
RMSD bond angles (°)	0.78	0.94	0.98	0.75
Estimated overall coordinate error based on Luzzati plot (Å)	0.25	0.28	0.37	1.33
PDB ID	8AVH	8AVI	8C3M	8C16

**Table 2:** Kinetic Parameters of Fld and NrdI Reduction by *Bc* FNR1-2 and mutants.

	FNR1 ^a,b^			FNR1V329H			FNR2 ^a,b^			FNR2H326V		
	*k*_cat_ (min^−1^)	*K*_M_ (μM)	*k*_cat_/*K*_M_ (μM^−1^ min^−1^)	*k*_cat_ (min^−1^)	*K*_M_ (μM)	*k*_cat_/*K*_M_ (μM^−1^ min^−1^)	*k*_cat_ (min^−1^)	*K*_M_ (μM)	*k*_cat_/*K*_M_ (μM^−1^ min^−1^)	*k*_cat_ (min^−1^)	*K*_M_ (μM)	*k*_cat_/*K*_M_ (μM^−1^ min^−1^)
**NrdI**	8.0 ± 0.1	2.7 ± 0.2	3.0 ± 0.2	1.8 ± 0.2	4.0 ± 1.3	0.5 ± 0.2	100 ± 4	61 ± 5	1.6 ± 0.2	36 ± 3	64 ± 9	0.6 ± 0.1
**Fld1**	7.3 ± 0.5	4.7 ± 0.8	1.5 ± 0.3	n.a.	n.a.	n.a.	2778 ± 401	25 ± 7	111 ± 47	183 ± 31	43 ± 11	4 ± 2
**Fld2**	42 ± 8	60 ± 22	0.7 ± 0.4	19 ± 6	12 ± 7	1.5 ± 1.0	9125 ± 1450	13 ± 5	701 ± 360	n.a.	n.a.	n.a.

^a^ Parameters for the reduction of NrdI by FNR1 and FNR2 are taken from [8]. ^b^ Parameters for the reduction of Fld1 and Fld2 by FNR1 and FNR2 are taken from [9]. n.a. indicates not available.

## Data Availability

The diffraction data for FNR2V326H and FNR1H329V collected at the ESRF, Grenoble, France, were archived in the ESRF depository (DOI: 10.15151/ESRF-DC-1183031629). X-ray coordinates and structure factors have been deposited in the Protein Data Bank (PDB) as entries 8AVH (*Bc* IsdG_apo_), 8AVI (*Bc* IsdG_heme_), 8C3M (*Bc* FNR1V329H), and 8C16 (*Bc* FNR2H326V) (PDB DOI: 10.2210/pdb8AVH/pdb, 10.2210/pdb8AVI/pdb, 10.2210/pdb8C3M/pdb, and 10.2210/pdb8C16/pdb).

## References

[1] Lu J., Holmgren A. (2014). The thioredoxin antioxidant system. Free Radic. Biol. Med..

[2] Macheroux P., Kappes B., Ealick S.E. (2011). Flavogenomics—A genomic and structural view of flavin-dependent proteins. FEBS J..

[3] Vidal L.S., Kelly C.L., Mordaka P.M., Heap J.T. (2018). Review of NAD(P)H-dependent oxidoreductases: Properties, engineering and application. Biochim. Biophys. Acta Proteins Proteom..

[4] Hammerstad M., Hersleth H.P. (2021). Overview of structurally homologous flavoprotein oxidoreductases containing the low *M*_r_ thioredoxin reductase-like fold—A functionally diverse group. Arch. Biochem. Biophys..

[5] Cotruvo J.A., Stubbe J. (2010). An active dimanganese(III)-tyrosyl radical cofactor in *Escherichia coli* class Ib ribonucleotide reductase. Biochemistry.

[6] Cotruvo J.A., Stubbe J. (2011). *Escherichia coli* class Ib ribonucleotide reductase contains a dimanganese(III)-tyrosyl radical cofactor in vivo. Biochemistry.

[7] Boal A.K., Cotruvo J.A., Stubbe J., Rosenzweig A.C. (2010). Structural basis for activation of class Ib ribonucleotide reductase. Science.

[8] Lofstad M., Gudim I., Hammerstad M., Røhr Å.K., Hersleth H.-P. (2016). Activation of the class Ib ribonucleotide reductase by a flavodoxin reductase in *Bacillus cereus*. Biochemistry.

[9] Gudim I., Hammerstad M., Lofstad M., Hersleth H.-P. (2018). The characterization of different flavodoxin reductase-flavodoxin (FNR-Fld) interactions reveals an efficient FNR-Fld redox pair and identifies a novel FNR subclass. Biochemistry.

[10] Hammerstad M., Gudim I., Hersleth H.-P. (2020). The crystal structures of bacillithiol disulfide reductase Bdr (YpdA) provide structural and functional insight into a new type of FAD-containing NADPH-dependent oxidoreductase. Biochemistry.

[11] Linzner N., Loi V.V., Fritsch V.N., Tung Q.N., Stenzel S., Wirtz M., Hell R., Hamilton C.J., Tedin K., Fulde M. (2019). *Staphylococcus aureus* uses the bacilliredoxin (BrxAB)/bacillithiol disulfide reductase (YpdA) redox pathway to defend against oxidative stress under infections. Front. Microbiol..

[12] Muraki N., Seo D., Shiba T., Sakurai T., Kurisu G. (2010). Asymmetric dimeric structure of ferredoxin-NAD(P)(+) oxidoreductase from the green sulfur bacterium *Chlorobaculum tepidum*: Implications for binding ferredoxin and NADP(+). J. Mol. Biol..

[13] Komori H., Seo D., Sakurai T., Higuchi Y. (2010). Crystal structure analysis of *Bacillus subtilis* ferredoxin-NADP(+) oxidoreductase and the structural basis for its substrate selectivity. Protein Sci..

[14] Loutet S.A., Kobylarz M.J., Chau C.H.T., Murphy M.E.P. (2013). IruO Is a reductase for heme degradation by IsdI and IsdG proteins in *Staphylococcus aureus*. J. Biol. Chem..

[15] Kobylarz M.J., Heieis G.A., Loutet S.A., Murphy M.E.P. (2017). Iron uptake oxidoreductase (IruO) uses a flavin adenine dinucleotide semiquinone intermediate for iron-siderophore reduction. ACS Chem. Biol..

[16] Reniere M.L., Ukpabi G.N., Harry S.R., Stec D.F., Krull R., Wright D.W., Bachmann B.O., Murphy M.E., Skaar E.P. (2010). The IsdG-family of haem oxygenases degrades haem to a novel chromophore. Mol. Microbiol..

[17] Gudim I., Lofstad M., van Beek W., Hersleth H.P. (2018). High-resolution crystal structures reveal a mixture of conformers of the Gly61-Asp62 peptide bond in an oxidized flavodoxin from *Bacillus cereus*. Protein Sci..

[18] Hammerstad M., Røhr Å.K., Hersleth H.P. (2019). A Research-inspired biochemistry laboratory module-combining expression, purification, crystallization, structure-solving, and characterization of a flavodoxin-like protein. Biochem. Mol. Biol. Educ..

[19] Skråmo S., Hersleth H.P., Hammerstad M., Andersson K.K., Røhr Å.K. (2014). Cloning, expression, purification, crystallization and preliminary X-ray diffraction analysis of a ferredoxin/flavodoxin-NADP(H) oxidoreductase (Bc0385) from *Bacillus cereus*. Acta Crystallogr. Sect. F Struct. Biol. Commun..

[20] Wang Z.Q., Lawson R.J., Buddha M.R., Wei C.C., Crane B.R., Munro A.W., Stuehr D.J. (2007). Bacterial flavodoxins support nitric oxide production by *Bacillus subtilis* nitric-oxide synthase. J. Biol. Chem..

[21] Gasteiger E., Gattiker A., Hoogland C., Ivanyi I., Appel R.D., Bairoch A. (2003). ExPASy: The proteomics server for in-depth protein knowledge and analysis. Nucleic Acids Res..

[22] Skaar E.P., Gaspar A.H., Schneewind O. (2006). *Bacillus anthracis* IsdG, a heme-degrading monooxygenase. J. Bacteriol..

[23] Vonrhein C., Flensburg C., Keller P., Sharff A., Smart O., Paciorek W., Womack T., Bricogne G. (2011). Data processing and analysis with the autoPROC toolbox. Acta Crystallogr. Sect. D Biol. Crystallogr..

[24] Kabsch W. (2010). XDS. Acta Crystallogr. Sect. D Biol. Crystallogr..

[25] Winn M.D., Ballard C.C., Cowtan K.D., Dodson E.J., Emsley P., Evans P.R., Keegan R.M., Krissinel E.B., Leslie A.G.W., McCoy A. (2011). Overview of the CCP4 suite and current developments. Acta Crystallogr. Sect. D Biol. Crystallogr..

[26] McCoy A.J., Grosse-Kunstleve R.W., Adams P.D., Winn M.D., Storoni L.C., Read R.J. (2007). Phaser crystallographic software. J. Appl. Crystallogr..

[27] Murshudov G.N., Skubak P., Lebedev A.A., Pannu N.S., Steiner R.A., Nicholls R.A., Winn M.D., Long F., Vagin A.A. (2011). REFMAC5 for the refinement of macromolecular crystal structures. Acta Crystallogr. Sect. D Biol. Crystallogr..

[28] Afonine P.V., Grosse-Kunstleve R.W., Echols N., Headd J.J., Moriarty N.W., Mustyakimov M., Terwilliger T.C., Urzhumtsev A., Zwart P.H., Adams P.D. (2012). Towards automated crystallographic structure refinement with phenix.refine. Acta Crystallogr. Sect. D Biol. Crystallogr..

[29] Adams P.D., Afonine P.V., Bunkoczi G., Chen V.B., Davis I.W., Echols N., Headd J.J., Hung L.W., Kapral G.J., Grosse-Kunstleve R.W. (2010). PHENIX: A comprehensive Python-based system for macromolecular structure solution. Acta Crystallogr. Sect. D Biol. Crystallogr..

[30] Emsley P., Lohkamp B., Scott W.G., Cowtan K. (2010). Features and development of Coot. Acta Crystallogr. Sect. D Biol. Crystallogr..

[31] Joosten R.P., Long F., Murshudov G.N., Perrakis A. (2014). The PDB_REDO server for macromolecular structure model optimization. IUCrJ.

[32] Chen V.B., Arendall W.B., Headd J.J., Keedy D.A., Immormino R.M., Kapral G.J., Murray L.W., Richardson J.S., Richardson D.C. (2010). MolProbity: All-atom structure validation for macromolecular crystallography. Acta Crystallogr. Sect. D Biol. Crystallogr..

[33] Zeldin O.B., Gerstel M., Garman E.F. (2013). RADDOSE-3D: Time- and space-resolved modelling of dose in macromolecular crystallography. J. Appl. Crystallogr..

[34] Zallot R., Oberg N., Gerlt J.A. (2019). The EFI web resource for genomic enzymology tools: Leveraging protein, genome, and metagenome databases to discover novel enzymes and metabolic pathways. Biochemistry.

[35] Shannon P., Markiel A., Ozier O., Baliga N.S., Wang J.T., Ramage D., Amin N., Schwikowski B., Ideker T. (2003). Cytoscape: A software environment for integrated models of biomolecular interaction networks. Genome Res..

[36] Sievers F., Wilm A., Dineen D., Gibson T.J., Karplus K., Li W.Z., Lopez R., McWilliam H., Remmert M., Soding J. (2011). Fast, scalable generation of high-quality protein multiple sequence alignments using Clustal Omega. Mol. Syst. Biol..

[37] Waterhouse A.M., Procter J.B., Martin D.M.A., Clamp M., Barton G.J. (2009). Jalview Version 2-a multiple sequence alignment editor and analysis workbench. Bioinformatics.

[38] Crooks G.E., Hon G., Chandonia J.M., Brenner S.E. (2004). WebLogo: A sequence logo generator. Genome Res..

[39] Novichkov P.S., Kazakov A.E., Ravcheev D.A., Leyn S.A., Kovaleva G.Y., Sutormin R.A., Kazanov M.D., Riehl W., Arkin A.P., Dubchak I. (2013). RegPrecise 3.0-A resource for genome-scale exploration of transcriptional regulation in bacteria. BMC Genomics.

[40] Desta I.T., Porter K.A., Xia B., Kozakov D., Vajda S. (2020). Performance and its limits in rigid body protein-protein docking. Structure.

[41] Vajda S., Yueh C., Beglov D., Bohnuud T., Mottarella S.E., Xia B., Hall D.R., Kozakov D. (2017). New additions to the ClusPro server motivated by CAPRI. Proteins.

[42] Kozakov D., Hall D.R., Xia B., Porter K.A., Padhorny D., Yueh C., Beglov D., Vajda S. (2017). The ClusPro web server for protein-protein docking. Nat. Protoc..

[43] Kozakov D., Beglov D., Bohnuud T., Mottarella S.E., Xia B., Hall D.R., Vajda S. (2013). How good is automated protein docking?. Proteins.

[44] Christoffer C., Chen S.Y., Bharadwaj V., Aderinwale T., Kumar V., Hormati M., Kihara D. (2021). LZerD webserver for pairwise and multiple protein-protein docking. Nucleic Acids Res..

[45] Christoffer C., Bharadwaj V., Luu R., Kihara D. (2021). LZerD protein-protein docking webserver enhanced with de novo structure prediction. Front. Mol. Biosci..

[46] Wu R.Y., Skaar E.P., Zhang R.G., Joachimiak G., Gornicki P., Schneewind O., Joachimiak A. (2005). *Staphylococcus aureus* IsdG and IsdI, heme-degrading enzymes with structural similarity to monooxygenases. J. Biol. Chem..

[47] Schuelke-Sanchez A.E., Cornetta A.R., Kocian T.A.J., Conger M.A., Liptak M.D. (2022). Ruffling is essential for *Staphylococcus aureus* IsdG-catalyzed degradation of heme to staphylobilin. J. Inorg. Biochem..

[48] Lee W.C., Reniere M.L., Skaar E.P., Murphy M.E.P. (2008). Ruffling of metalloporphyrins bound to IsdG and IsdI, two heme-degrading enzymes in *Staphylococcus aureus*. J. Biol. Chem..

[49] Meschi F., Wiertz F., Klauss L., Blok A., Ludwig B., Merli A., Heering H.A., Rossi G.L., Ubbink M. (2011). Efficient electron transfer in a protein network lacking specific interactions. J. Am. Chem. Soc..

[50] Pinochet-Barros A., Helmann J.D. (2018). Redox sensing by Fe^2+^ in bacterial Fur family metalloregulators. Antioxid. Redox Signal..

[51] Chandrangsu P., Rensing C., Helmann J.D. (2017). Metal homeostasis and resistance in bacteria. Nat. Rev. Microbiol..

[52] Baichoo N., Wang T., Ye R., Helmann J.D. (2002). Global analysis of the *Bacillus subtilis* Fur regulon and the iron starvation stimulon. Mol. Microbiol..

[53] Baichoo N., Helmann J.D. (2002). Recognition of DNA by Fur: A reinterpretation of the Fur box consensus sequence. J. Bacteriol..

[54] Hayrapetyan H., Siezen R., Abee T., Groot M.N. (2016). Comparative genomics of iron-transporting systems in *Bacillus cereus* strains and impact of iron sources on growth and biofilm formation. Front. Microbiol..

[55] Pi H.L., Helmann J.D. (2017). Sequential induction of Fur-regulated genes in response to iron limitation in *Bacillus subtilis*. Proc. Natl. Acad. Sci. USA.

[56] Skaar E.P., Gaspar A.H., Schneewind O. (2004). IsdG and IsdI, heme-degrading enzymes in the cytoplasm of *Staphylococcus aureus*. J. Biol. Chem..

[57] Skaar E.P., Schneewind O. (2004). Iron-regulated surface determinants (Isd) of *Staphylococcus aureus*: Stealing iron from heme. Microbes Infect..

[58] Sevilla E., Bes M.T., Peleato M.L., Fillat M.F. (2021). Fur-like proteins: Beyond the ferric uptake regulator (Fur) paralog. Arch. Biochem. Biophys..

[59] Seo D., Muraki N., Kurisu G. (2020). Kinetic and structural insight into a role of the *re*-face Tyr328 residue of the homodimer type ferredoxin-NADP(+) oxidoreductase from *Rhodopseudomonas palustris* in the reaction with NADP(+)/NADPH. Biochim. Biophys. Acta Bioenergetics..

[60] Bashir Q., Scanu S., Ubbink M. (2011). Dynamics in electron transfer protein complexes. FEBS J..

[61] Seo D., Asano T. (2018). C-terminal residues of ferredoxin-NAD(P)(+) reductase from *Chlorobaculum tepidum* are responsible for reaction dynamics in the hydride transfer and redox equilibria with NADP(+)/NADPH. Photosynth. Res..

[62] Seo D., Asano T., Komori H., Sakurai T. (2014). Role of the C-terminal extension stacked on the *re*-face of the isoalloxazine ring moiety of the flavin adenine dinucleotide prosthetic group in ferredoxin-NADP(+) oxidoreductase from *Bacillus subtilis*. Plant Physiol. Biochem..

[63] Waksman G., Krishna T.S.R., Williams C.H., Kuriyan J. (1994). Crystal structure of *Escherichia-coli* thioredoxin reductase refined at 2-Ångström resolution—Implications for a large conformational change during catalysis. J. Mol. Biol..

[64] Shoor M., Gudim I., Hersleth H.P., Hammerstad M. (2021). Thioredoxin reductase from *Bacillus cereus* exhibits distinct reduction and NADPH-binding properties. FEBS Open Bio.

